# One-on-one and group-based physical activity intervention compared to a waitlist control for post-secondary student mental health and social well-being: A 3-arm parallel randomized controlled trial protocol

**DOI:** 10.1371/journal.pone.0330851

**Published:** 2025-08-29

**Authors:** Melissa L. deJonge, Sandra Yuen, Luc Simard, Catherine M. Sabiston

**Affiliations:** 1 Faculty of Kinesiology and Physical Education, University of Toronto, Toronto, Ontario, Canada; 2 Department of Psychology, University of Toronto, Toronto, Ontario, Canada; Universidade Estadual de Santa Cruz, BRAZIL

## Abstract

**Introduction:**

Physical activity (PA) service provision in the post-secondary context is recognized as important for promoting student mental health. Nonetheless, most evidence is of poor quality and lacks critical information regarding how the PA programs are designed, delivered, and made accessible to students. This study will examine PA program effectiveness for student mental health and social well-being, as well as implementation processes to offer insight for future research and program scale-up.

**Methods and analysis:**

Post-secondary students who are physically inactive and experiencing poor mental health will be recruited. A 3-arm parallel Randomized Controlled Trial, using a hybrid effectiveness-implementation design, will be conducted using a collaborative implementation approach. The effects of 6-week supervised one-on-one and group PA, compared to a waitlist control will be examined, with outcomes assessed at baseline (T1), 6-weeks (T2), and 1-month follow-up (T3). Primary outcomes will include immediate post-program changes (T1–T2) in mental health indices, including anxiety, depression, psychological distress, and well-being. Secondary outcomes will include changes from baseline to follow-up (T1–T3) and maintenance effects from post-intervention to follow-up (T2–T3) in mental health indices, as well as changes in social well-being indices (i.e., social connectedness, social support), and PA behavior. A process evaluation will be conducted to explore contextual influences (i.e., fidelity, adherence, reach, acceptability) on the conduct of implementation across PA program delivery styles. Effectiveness data will be analyzed using linear mixed effects modeling. Process evaluation outcomes will be analyzed using a mixed methods evaluation.

**Dissemination:**

A knowledge mobilization plan to enhance dissemination of the findings to the intended audiences (i.e., sport and recreation professionals, mental health professionals, students, researchers) has been developed.

**Trial registration:**

ClinicalTrials.gov NCT06350877

## Introduction

For reasons related to the educational context (e.g., academic and financial pressures) and life-span characteristics (e.g., identity exploration, instability), post-secondary students are a vulnerable population for experiencing mental health concerns [1]. Providing targeted services for supporting post-secondary student mental health is critical for promoting adaptive trajectories of functioning, adjustment, and well-being across the lifespan [2]. Mental health services offered on post-secondary campuses vary in range and support including promotion and outreach programs, short-term therapy, crisis appointments, and social or peer support programs [3,4]. Despite the services available to students, barriers to help-seeking are widely reported [5,6]. Indeed, less than half of students who experience poor mental health are receptive to traditional mental health services (e.g., medication counseling), with many students preferring alternative or complementary services such as those focused on lifestyle [7,8]. As a result, research focused on the implementation of sustainable approaches for offering alternative and complementary mental health services on post-secondary campuses has critical implications for supporting students who may not access traditional services.

PA is one alternative and complementary treatment approach that could be widely offered as an evidence-based approach for supporting student mental and physical health [9–11]. In support of PA as an alternative and complementary treatment, there has been substantial endorsement of major guidelines supporting the use of PA for the prevention and treatment of mental health conditions across clinical and non-clinical populations [12]. Considering the strong support for PA in the prevention and treatment of various mental health conditions, research focused on enhancing the provision of structured and tailored PA programs for post-secondary student mental health is increasing and promising [13–15]. Yet, there are notable knowledge gaps and study design limitations, which are suggested to contribute to the poor translation of accessible and sustainable PA programs tailored toward student mental health in the post-secondary community [13,16].

First, research to date has predominantly summarized single-group designs with a lack of a control group and randomization. This contributes to limitations in the confidence and quality of the implications drawn from the synthesized studies. Second, there is a paucity of research exploring the effects of different delivery styles (i.e., one-on-one (1:1) vs. group) on primary (i.e., mental health symptomology reduction) and secondary (i.e., social support, social connectedness) outcomes. Group-based PA, in comparison to 1:1 delivered PA, may provide a less costly and less resource intensive intervention option, and may have unique benefits associated with exercising with others and peer-to-peer support [17,18]. Drawing on self-determination theory [19] and the social identity approach to health [20], group-based PA may activate psychosocial mechanisms (e.g., relatedness, social cohesion) that are particularly relevant for promoting mental health and well-being. Third, the maintenance effects of PA programs on mental health or sustained PA behavior change are largely unknown. As such, conclusions of achieving lasting change to mental health and sustained PA involvement are not possible. Lastly, researchers have predominantly focused on effectiveness outcomes (i.e., testing effects on relevant outcomes) with limited to no research exploring implementation outcomes (i.e., the practical aspects of delivering a program). The limited research on implementation outcomes precludes understanding how to implement successful programs that are accessible for students with poor mental health in the post-secondary community [16,21]. To improve our understanding of implementation outcomes and the translation of research into practice, hybrid effectiveness-implementation studies which integrate both effectiveness and implementation stages of intervention development are recognized as important [22,23].

### Objectives and hypotheses

Using a type 1 hybrid effectiveness-implementation study design [22], this three-arm parallel randomized controlled trial (RCT) will evaluate the effects of 1:1 and group-based supervised PA, compared to a 10-week waitlist control, among post-secondary students across three timepoints: baseline (T1), post-intervention (T2; 6-weeks), and follow-up (T3; 1-month post-intervention). Primary outcomes will include the immediate changes (T1–T2) in mental health indices (i.e., anxiety, depression, psychological distress, and well-being). Secondary outcomes will include changes from baseline to follow-up (T1–T3) and maintenance effects from post-intervention to follow-up (T2–T3) in mental health indices, as well changes in social well-being indices (i.e., social connectedness, social support), and PA behavior. The aims of the study include: (1) examining group differences between 1:1 PA delivery, group-based PA delivery, and the 10-week waitlist control arm on the primary and secondary outcomes; and (2) grounded in process evaluation recommendations [24], to explore implementation outcomes (i.e.,., reach, adherence, acceptability, fidelity) that may be linked to variation in primary and secondary outcomes while offering insight for wider dissemination. Based on evidence supporting various PA modalities for improving mental health [25], both 1:1 and group-based PA are hypothesized to be more effective than the waitlist control on primary and secondary outcomes, with no expected differences between the PA conditions on primary outcomes. Drawing on the social identity approach to health [20], it is hypothesized that group-based delivery will result in greater improvements in secondary outcomes including social well-being outcomes and maintenance effects compared to 1:1 delivery. However, 1:1 delivery is expected to yield more favourable implementation outcomes, due to its potential for stronger individualized support [14,26]. The results will provide novel insight into the effectiveness of different PA program delivery styles on student mental health, social well-being, and PA behaviour, while also offering implementation considerations to support the sustainability and scale-up of PA interventions across post-secondary campuses.

## Methods

### Trial design

A 3-arm parallel RCT assessing the intervention arms (1:1 and group-based PA delivery) compared to a 10-week control arm (waitlist control) will be conducted. A parallel arm design will be implemented, whereby students will be randomized to a study arm, and each study arm will be allocated a different intervention. The protocol adheres to CONSORT guidelines [27] and SPIRIT [28] recommendations for reporting of clinical trial protocols (see S1 Checklist). Ethical approval for the study was obtained November 24, 2023 (protocol # 45228) and the study was retrospectively registered on ClinicalTrials.gov (Study identifier: NCT06350877; Registered: April 2, 2024). The clinical trial was retrospectively registered due to the initial phase of data collection being focused on piloting and refining the data collection methods.

### Study setting

The trial will leverage the infrastructure and support provided by Sport and Recreation Services, Health and Wellness Services, and the Mental Health and Physical Activity Research Centre at the University of Toronto’s St. George campus. A collaborative implementation approach involving the knowledge-user groups (i.e., researchers, professionals in on-campus sport and recreation and mental health, students) will be used to gather insights on embedding tailored and structured PA programs for student mental health within existing sport and recreation, and mental health services on post-secondary campuses.

### Patients and public involvement

The study is informed by previous research on student perspectives of PA programming for mental health [29] and a mixed methods evaluation of the proposed PA intervention protocol, within a 1:1 delivery setting [14]. Student feedback will be collected throughout the study and used to inform insights for wider program dissemination and scale-up. Using a train-the-trainer model [30], the research team will collaborate with Sport and Recreation Services to provide standardized training in behavior change coaching and PA program delivery for mental health to certified student PA coaches. These trained and certified coaches will be responsible for delivering the PA intervention. Referral pathways from on-campus mental health and accessibility services will be examined. Consistent with type 1 hybrid effectiveness-implementation studies [22], process-related information on coach training and the referral pathways to the program will be collected to inform future testing and broader dissemination of educational tools and implementation guidelines. Input from on-campus mental health and sport and recreation professionals, as well as students, has informed the evaluation targets for the trial. Input emphasized the importance of examining the effectiveness of different PA delivery styles on broader well-being outcomes, such as social well-being. Knowledge-users (i.e., sport and recreation professionals, mental health professionals, students, researchers) will also be consulted during knowledge mobilization and dissemination to enhance the useability and uptake of the findings.

### Eligibility criteria

Post-secondary students will be recruited based on the following eligibility criteria: (a) a post-secondary undergraduate or graduate student enrolled either part-time or full-time; (b) fluent in English (e.g., proficiency in reading and verbal expression – written and oral); (c) able to attend in-person PA sessions at the campus athletics and recreation centre; (d) moderately or insufficiently active (≤ 23 units of weekly leisure activity) based on interpretation scores from the Godin Leisure-Time Exercise Questionnaire [GLTEQ; 31]; and (e) experiencing self-reported ‘poor’, ‘fair’ or ‘good’ mental health in the past month. Exclusion criteria will include: (a) physically active (24 units or more of weekly leisure activity); (b) unsuccessful PA clearance using the PA readiness questionnaire [PAR-Q^+^; 32]; and (c) self-reported ‘very good’ or ‘excellent’ mental health.

### Schedule of enrolment, interventions, and assessments

**Fig 1:**
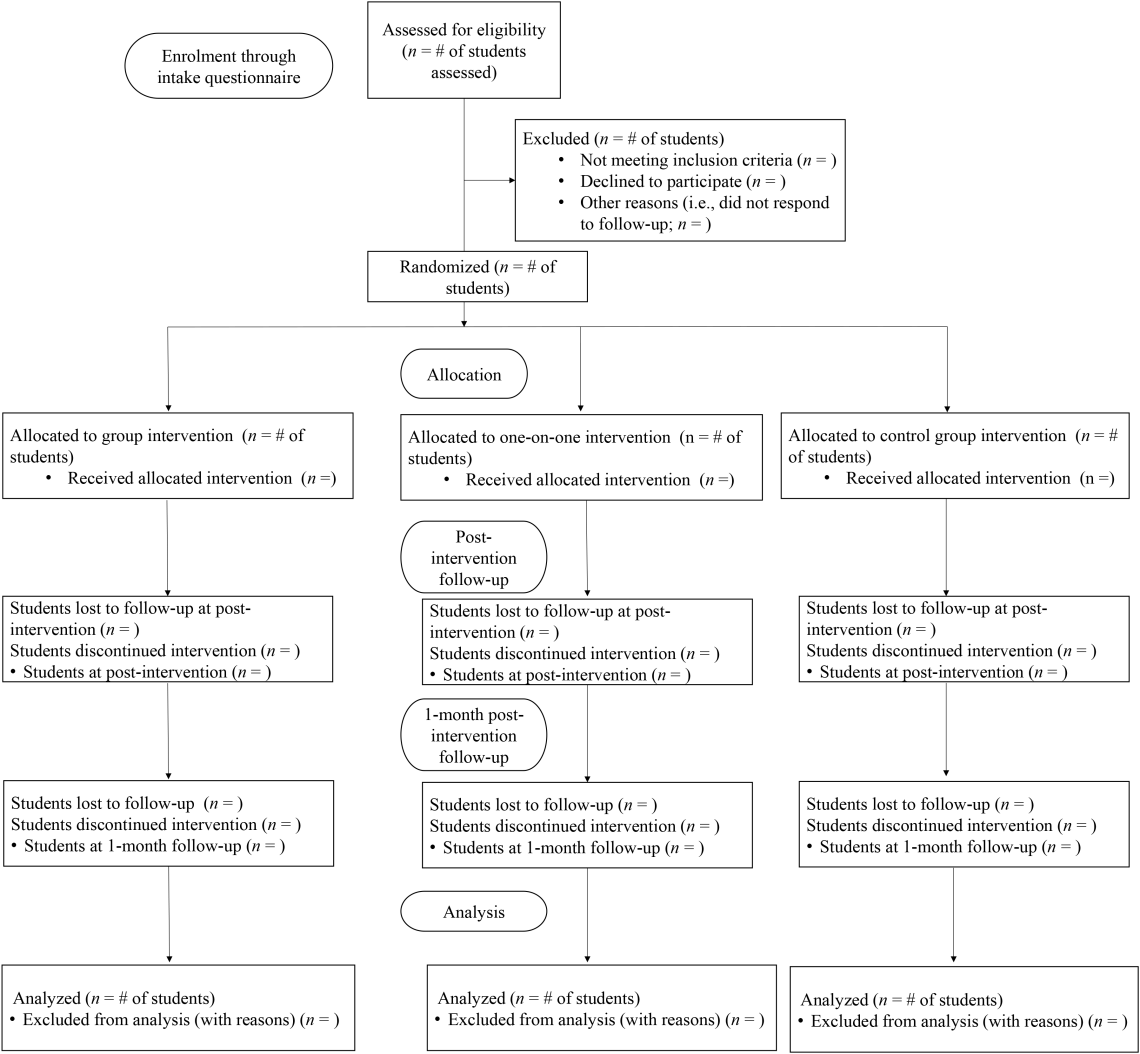
Participant flow.

**Fig 2:**
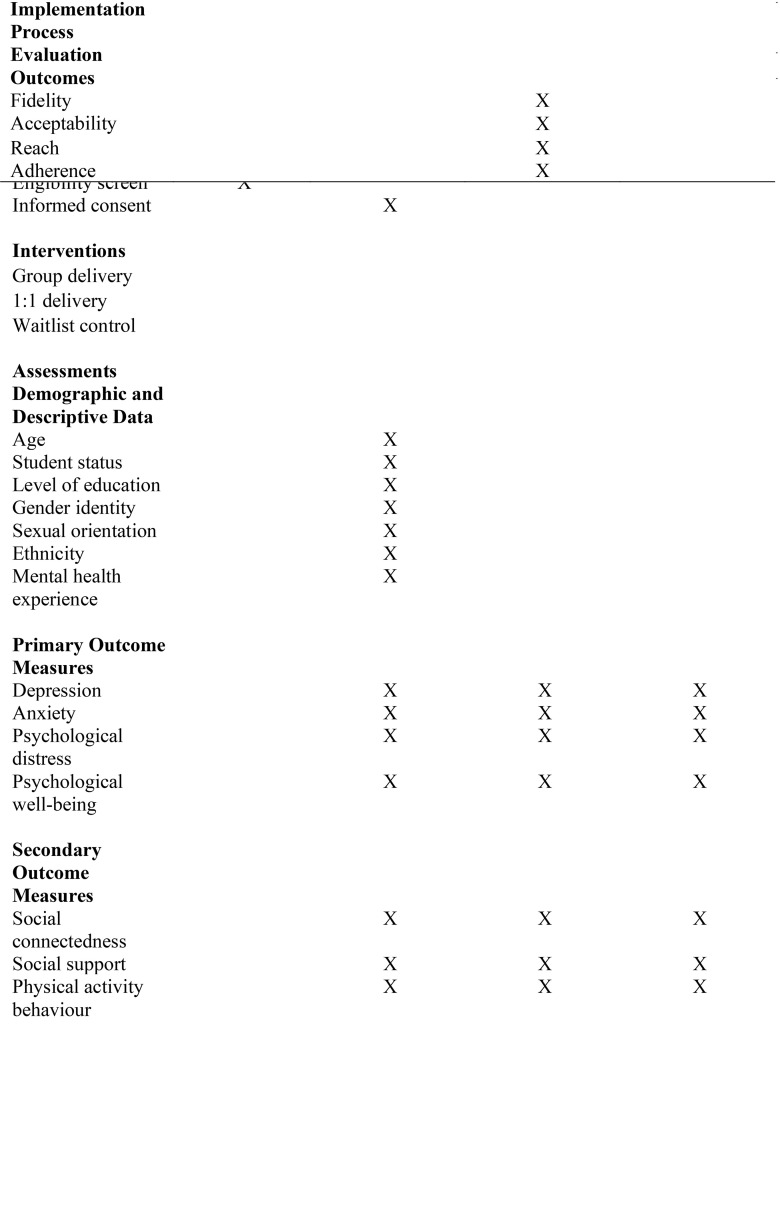
Schedule of enrolment, interventions, and assessments according to the Standard Protocol Items: Recommendations for Intervention Trials.

After screening for eligibility, students will be contacted to schedule an in-person intake session with a program coordinator for the trial to provide informed consent, complete the baseline assessment (T1), and conduct randomization. In the intervention arms and control arm, study outcomes will be assessed at baseline (T1), 6-weeks post baseline (T2), and at 1-month follow-up (T3). Aligning with SPIRIT [28] and CONSORT [27] guidelines intervention flow will be tracked (see Fig 1) and the schedule of enrolment, interventions, and assessments has been provided (see Fig 2). A 1-month follow-up was selected for the current study given that, to date, only one known study has examined the maintenance effects of PA interventions on student mental health outcomes [16]. The aim is to assess whether the 1-month effects observed in prior research can be replicated in the current context [33]. If such findings are observed, this follow-up period will provide an important foundation for future studies to evaluate the sustainability and longer-term impact of these interventions. To support student retention at T3, a modest financial incentive will be offered in the form of a $25 CAD gift card (student’s choice of Amazon, Indigo, or Starbucks). Students’ reasons for discontinuing the trial will be collected, no further data will be collected following withdrawal from the trial.

### Allocation and blinding

Simple randomization will be used to allocate eligible students to the intervention arms using Research Randomizer [www.randomizer.org; 34,35]. Each student will be individually randomized, with an equal chance of being assigned to any of the three groups (1:1 PA, group-based PA, or waitlist control). While simple randomization may lead to unequal group sizes, block randomization was deemed unsuitable due to the risk of generating groups not comparable on covariates [34]. Stratified randomization was not possible given the continuous nature of student enrollment [34]. To account for the potential group size imbalance, primary statistical analyses will use linear mixed effects modeling, which are robust to unequal sample sizes and unbalanced data [36].

To minimize selection bias, allocation concealment will be used by having an investigator who will generate and assign random group allocations only after students have completed enrollment and the baseline (T1) assessment. Random allocations will be generated in real-time and not stored in advance. Students will be partially blinded in that students will be unaware of the purpose of group allocation or study hypotheses but will know they were assigned to an intervention arm or the control arm. To prevent care provider bias, PA coaches from Sport and Recreation Services will also not be informed about the purpose of group allocation or the study hypotheses. Investigators will not be blinded due to their roles in program coordination and statistical analyses. Recruiting an external individual to manage randomization, program coordination, and analyses is not feasible given the scope of the study and availability of resources. Nonetheless, several steps will be taken to minimize bias. Allocation concealment will be maintained until after baseline assessment, and analyses will follow the pre-specified plan outlined in this protocol paper. Outcome measures include open-ended and standardized self-report items, completed by students through a secure online data capture platform [REDCap; 37] without direct investigator involvement. The study protocol is also pre-registered to support transparency and reduce the risk of selective reporting.

### Trial arms

Eligibility screening and randomization to trial arms will occur on a rolling basis, aligned with designated recruitment periods in the fall, spring, and summer semesters. All PA sessions will be delivered by trained Sport and Recreation PA coaches at the University of Toronto. Following group allocation to an intervention arm (1:1 or group-based PA), students will be matched with a coach and work with the same coach throughout the duration of the 6-week program. All PA sessions for the intervention arm are expected to commence within one week of randomization. Students assigned to 1:1 delivery will be matched immediately with a PA coach to coordinate weekly sessions. Group-based PA sessions will start once 3–8 students have been allocated and will be scheduled at a time that works for most students enrolled. Modifications to the allocated trial arm (e.g., switching from group to 1:1 PA delivery or from the control arm to an intervention arm) will not be permitted within the research protocol. However, students who are unable to follow through with their assigned trial arm will be offered PA sessions outside of the research (1:1 or group-based, depending on student preference) as part of standard service provision. Reasons for not initiating an assigned trial arm will be documented as part of the mixed methods process evaluation, as an indicator of adherence to the trial. To improve adherence to the intervention protocol by students, coaches will be in contact with students weekly to schedule sessions and will send reminder emails to students about upcoming sessions. To improve adherence to the intervention protocol by coaches, a standardized behavior change coaching booklet will be used to guide all PA intervention sessions. Regular bi-weekly check-ins with PA coaches and the research team will be conducted to support consistent delivery and to monitor implementation and student progression through the PA intervention. In addition, fidelity to core intervention processes will be examined based on student-reported experiences (see Implementation Process Evaluation Outcomes). Concomitant mental health care (e.g., therapy, medication use) and engagement in additional on-campus resources will be permitted during the trial and reported on to describe the student sample.

### Intervention arms

The intervention arms are grounded in behavior change theory and empirical evidence on PA for mental health [14,25,38,39], with sessions designed to meet students’ PA related needs and preferences. To support student preferences, PA sessions will be delivered in-person in private PA spaces in the Mental Health and Physical Activity Research Center, located in the campus athletics and recreation centre. Public gym spaces will also be used depending on student preferences and interests. While bias related to coaching variability and differences in PA delivery space is plausible, the intervention was intentionally designed using pragmatic principles to reflect real-world conditions [40]. This includes allowing flexibility in implementation, such as permitting coaches with diverse backgrounds to deliver the sessions and enabling students to select preference-based PA delivery space. The purpose is to facilitate enjoyment and key COM-B behavioral processes (capability, opportunity, and motivation), to support ongoing engagement in PA as a mental health management strategy (see Table 1). Indeed, the COM-B model posits that no behavior will occur without sufficient capability, opportunity, and motivation [43]. The mixed methods process evaluation was designed to assess whether core intervention processes are received by students including acceptability (e.g., enjoyment), student-reported experiences of COM-B processes, and the quality of the coach-student relationship, a core component intended to foster a supportive and collaborative environment.

**Table 1:** Intervention core components using the COM-B model and measurement items for implementation fidelity.

COM-B constructs	Description	Measurement items
		**Stem:** My PA coach…
**Capability**	• Enhancing psychological capability to reduce barriers to engaging in PA. For example, action planning and problem solving to ensure students feel supported to be active outside of the supervised PA session • Enhancing physical capability to engage in PA. For example, demonstrating correct PA techniques and providing individualized and personalized PA considerations	“Took into account my needs and preferences” “Was knowledge about PA” “Demonstrated PA techniques (e.g., proper form) in a way that improved my confidence and capability” “Explored options with me to find something I would enjoy”
**Opportunity**	• Considering environmental and social opportunities for supporting engagement in PA – including providing a supportive and enjoyable PA environment	“Provided a supportive environment” “Helped support me to be active outside of the MoveU.HappyU sessions” “Provided an enjoyable PA experience”
		“Arranged session times that suited me”
		“Was easily contactable”
**Motivation**	• Enhancing motivation through targeting knowledge and education around the benefits of PA for mental health • Enhancing motivation through feedback and monitoring strategies such as goal setting and encouraging habit formation through prompts and cues • Eliciting positive emotions through emotional social support	“Helped my motivation for PA” “Provided me with information about things that may influence my mental health” “Was caring”
		“Was someone I liked”

*Note.* PA = physical activity; Measurement items were informed by recommendations for applying the COM-B model to physical activity intervention design and empirical evidence [41,42].

### 1:1 PA intervention delivery

The 1:1 PA intervention will be a 6-week supervised and individualized program. Participation will involve engaging in a weekly 1-h session. Each 1-h session will include: (1) 30-min of behavior change coaching; and (2) 30-min of individualized and supervised PA training. Intervention material includes a behavior change workbook for facilitating the 30-min of behavior change coaching, which has been informed by evidence-based strategies for promoting mental health through PA engagement [39]. Each week, students will complete the behavior change workbook in session with the program trainer to support the weekly learning objectives and learning experiences (see Table 2). Following completion of the 30-minute behavior change coaching, students will engage in structured and supervised PA. For a detailed description of the PA intervention protocol see [14].

**Table 2:** Weekly behavior change coaching learning objectives, learning experiences, and associated behavior change techniques.

Session	Behavior change techniques	Learning objectives and experiences
**Session 1: Getting started**	• Behavioral contract • Commitment • Monitoring of emotional consequences^+^	To facilitate greater awareness of current PA behavior and reasons/motives for wanting to become more physically active To affirm commitment to PA behavior change using a behavioral contract To introduce the feeling scale [44] and felt arousal scale [45] as a strategy for monitoring the emotional consequences of engaging in PA
**Session 2: Goal setting**	• Goal setting (behavior)^+^ • Action planning^+^ • Self-monitoring of behavior^+^	To become familiar with the benefits and principles of goal setting following recommendations for PA prescription and referral [46,47] To apply principles of goal setting to PA behavior change [48] To learn how to use the RPE scale [49] as a strategy to self-monitor exertion during PA and to set intensity goals (i.e., to increase or decrease intensity to reach the desired intensity range)
**Session 3: Self-talk & Self-compassion**	• Review behavior goal(s)^+^ • Discrepancy between current behavior and goal^+^ • Self-talk • Reduce negative emotions	To review behavior goals (including discussing modifying future goals) and to draw attention to any discrepancies between current behavior and the previously set goal To understand how self-talk (including inner commentary, self-statements, and thought traps) may be influencing PA progress To develop personally meaningful self-talk statements to encourage engagement in PA and to help feel good after being physically active To introduce self-compassion [50] as a strategy for managing negative self-talk and negative emotions to prevent interference with PA progress
**Session 4: Overcoming barriers to PA**	• Problem solving • Prompts/cues	To reflect on barriers to PA behavior change and problem solve solutions for overcoming barriers To introduce if-then planning [51] as a problem-solving strategy for overcoming barriers to PA behavior change To understand cues to action and personally meaningful prompt/cues (e.g., set an alarm, leave workout clothes by the door) in the environment to help facilitate PA behavior change
**Session 5: Maintaining motivation**	• Focus on past successes • Problem solving • Social support – unspecified, practical, or emotional depending on individual PA related needs	To understand personal fluctuations in motivation for PA engagement and to understand past successful strategies for maintaining current and future motivation To understand PA relapse prevention strategies and problem solve a relapse comeback plan To discuss social support as a strategy for maintaining motivation and to identify a social support network for facilitating PA engagement
**Session 6: Building on your tools**	• Problem solving	To reflect upon the strategies and tools learned throughout the intervention and to problem solve a plan for continuing to engage in PA following completion of the intervention

*Note.* PA = physical activity; RPE = rating of perceived exertion.

+ Behavior change technique reoccurs in each session.

For a detailed description of the behavior change techniques see [38].

### Group PA intervention delivery

The group PA intervention will receive the same protocol as described above though delivered in small groups of 3–8 students led by a trained Sport and Recreation PA coach. Drawing on group-dynamic principles [20,52], this number of students is ideal to help foster cohesiveness (e.g., through increased opportunity for interaction, discussion, and feedback/support from the PA coach) while reducing coordination and scheduling barriers. To promote needs satisfaction and preferences in group-based delivery, coaches will promote shared decision making on the types of activities engaged in within the group sessions.

### Control arm

Students who are assigned to the 10-week waitlist control arm will be assessed on primary and secondary outcomes at baseline (T1), at 6-weeks (T2), and at 1-month follow-up (T3). At completion of the 1-month follow-up assessment, students in the waitlist control arm will be offered the PA intervention (either group or 1:1 delivery depending on student preference). Following completion of the control arm, behavior will not be monitored or evaluated.

### Outcomes

Research outcomes to be assessed include demographic and descriptive data, primary outcomes, secondary outcomes, and implementation process evaluation outcomes.

### Demographic and descriptive data

Self-reported data to describe the characteristics of the sample will be collected including age in years, international student identification (i.e., domestic or international student), level of education (i.e., undergraduate, graduate, college program, either full-time or part-time), gender identity (i.e., women, non-binary, two-spirit, man, prefer not to answer), sexual orientation (i.e., heterosexual/straight, gay/lesbian, bisexual, pansexual, asexual, queer, two-spirit, questioning/unsure, I prefer not to answer, other), and ethnicity/race (i.e., Indigenous peoples of Canada, Indigenous outside of Canada, Arab, Black, Chinese, Filipino, Japanese, Korean, Central or South American, South Asian, Southeast Asian, West Asian, White). Students will also be asked to self-report their mental health experiences including history of mental illness using organizational structure from the DSM-5 [53], past month emotional challenges (i.e., loneliness, difficultly coping with stress in a healthy way, difficulty handling emotions, anxiety, social isolation, depression, trouble concentrating, substance use issues, unhealthy social media use), and past year mental health service use (i.e., past-year therapy or counseling, past year medication, therapy or counseling and medication use, not applicable, and other).

### Primary outcomes

**Depression.** The Patient Health Questionnaire [PHQ-9; 54] will be used. The 9-item PHQ measures the presence and severity of depressive symptoms over the past two weeks (e.g., “Feeling tired or having little energy”; “Little interest or pleasure in doing things”) ranging from 0 (*not at all*) to 3 (*nearly every day*) and aligns with the DSM-IV criteria to screen for and measure the severity of depression [54]. The total summed score ranging from 0−27 will be used in main analyses and depression symptom severity including minimal (score ranging from 5−9), minor (score ranging from 10−14), moderately severe (score ranging from 15−19) and severe (score > 20) will be calculated for descriptive purposes [54]. The PHQ-9 has high reliability and validity for screening and assessing the severity of depression and is widely used in both clinical practice and research including among post-secondary students [54–56].

**Anxiety.** The Generalized Anxiety Disorder Questionnaire [GAD-7; 57] will be used. The 7-item GAD assesses the frequency of symptoms associated with anxiety during the past two weeks (e.g., “Feeling nervous anxious or on edge”; “Trouble relaxing”) ranging from 0 (*not at all*) to 3 (*nearly every day*). The total summed score ranging from 0−21 will be used in main analyses and anxiety symptom severity including minimal (score ranging from 0−4), mild (score ranging from 5−9), moderate (score ranging from 10−14) and severe (score ranging from 15−21) will be calculated for descriptive purposes [57]. The GAD-7 is a valid and reliable measurement for assessing anxiety symptoms and the psychometric properties among young adults and post-secondary students have been demonstrated [57–59].

**Psychological distress.** The 10-item Kessler Psychological Distress Scale [K10; 60] will be used. Students will be asked to indicate how often over the last 30 days they experienced symptoms of psychological distress (e.g., “How often did you feel hopeless?”; “How often did you feel worthless?”) ranging from 1 (*none of the time*) to 5 (*all of the time*). The total summed score ranging from 10–50 will be used in analyses, with higher scores reflecting more psychological distress. Evidence of reliability and validity of the K10 has been reported among community and clinical samples [61,62] and is frequently used to monitor psychological distress among post-secondary students [63,64].

**Well-being.** The Mental Health Inventory-38 [MHI-38; 65] will be used to measure the 14-item psychological well-being subscale. Students will be asked to report how often during the past month they experienced symptoms of psychological well-being (e.g., “How much of the time, during the past month, did you feel relaxed and free from tension”; “During the past month, how much of the time have you generally enjoyed the things you do?”) on a six-point Likert scale ranging from 1 (*all of the time*) to 6 (*none of the time*). A total summed score will be used in main analyses ranging from 14–84, where higher scores represent more positive experiences of well-being. The MHI-38 has been widely used to measure well-being, and the psychometric properties have been demonstrated [66,67].

### Secondary outcomes

**Social support**. The Social Provision Scale [SPS-5; 68] will be used to measure social support. The scale consists of 5-items (e.g., “I have relationships where my competence and skill are recognized”; “I feel part of a group who share my attitudes and beliefs”), on a 4-point Likert scale ranging from 1 (*strongly disagree*) to 4 (*strongly agree)*. A total summed score ranging from 5–20 will be used in main analyses, where higher scores indicate more favorable perceptions of social support. The scales validation has been shown in the general population and the post-secondary student population [68].

**Social connectedness.** The 8-item Social Connectedness Scale will be used to measure social connectedness [69]. The items portray a general emotional distance between the self and others (e.g., “I feel disconnected from the world around me”; “I don’t feel I participate with anyone or any group”) and reflect behavior, feelings, or both associated with a lack of connectedness on a 6-point Likert scale ranging from 1 (*agree*) to 6 (*disagree*). Higher scores will reflect a more reported sense of social connectedness with a potential range of 8–48. The Social Connectedness Scale displays strong psychometric properties among post-secondary students [69].

**PA behavior.** Self-reported PA behavior will be measured using the GLTEQ [70]. Students will be asked to indicate how many times on average they engaged in mild (e.g., yoga, golf), moderate (e.g., fast walking, baseball), and vigorous (e.g., running, soccer) PA for more than 15 minutes in a typical week. A common modification of assessing the average duration (in hours and minutes) per session of each intensity category of PA will be included [71–73]. Students will also be asked to report the frequency and average duration of resistance exercise (e.g., free weight, bodyweight training). Total scores for mild, moderate, and vigorous activity and resistance exercise will be calculated by multiplying self-reported times per week by average duration of sessions for each type. The scale has been used among post-secondary students [73] and has been validated for use in adult samples [70,73].

### Implementation process evaluation outcomes

Informed by previous research [14,29], the mixed methods implementation evaluation plan (see Table 3) will assess intervention fidelity, acceptability, reach, and adherence. Intervention fidelity will assess the extent to which students perceive their coach adhered to core intervention targets guided by the COM-B model, as well as the extent to which the collaborative coach–student relationship supported task, goal, and bond components of working therapeutic alliance [74]. Program acceptability will be assessed based on students’ enjoyment, and their perceptions of the program’s effectiveness as a mental health intervention. In addition, motives for engagement, perceptions of the intervention content (i.e., what worked well and opportunities for improvement), and delivery format preferences (i.e., no preference, 1:1, or group-based) will be assessed to help inform future program refinement to improve acceptability. Reach will assess referral pathways, as well as program inclusion and exclusion considerations. Adherence will be assessed based on the number of students who initiated the intervention, the number of sessions completed, and overall student completion rates.

**Table 3:** Mixed methods implementation evaluation.

Implementation evaluation construct	Method, source of data, example items	
	**Quantitative data**	**Qualitative data**
**Intervention fidelity**		
COM-B core intervention components	Student perceptions of the integration of core COM-B targets by the PA coach across 13-items on a 7-point Likert scale ranging from 1 (*strongly disagree*) to 7 (*strongly agree*) will be used to assess intervention fidelity (see Table 1 for items).	N/A
Collaborative coach-student relationship	The 12-item Working Alliance Inventory will be used [WAI-12; 74]. Aligning with previous research [72], the WAI-12 will be adapted to the PA context (e.g., “My PA coach and I agree on what is more important for me to work on”) and students will be asked to report working alliance perceptions on a 7-point Likert scale ranging from 1 (*never*) to 7 (*always*).	N/A
**Acceptability**		
Enjoyment	3-items (e.g., “I enjoyed the exercise sessions very much”; “The exercise sessions were fun to do”) will be used on a 5-point Likert scale ranging from 1 (*strongly disagree*) to 5 (*strongly agree*).	N/A
Acceptability of the program as a mental health intervention	4-items (e.g., “The program is a useful component of on-campus mental health promotion”; “The sessions were good for my mood and general well-being”) will be used on a 5-point Likert scale ranging from 1 (*strongly disagree*) to 5 (*strongly agree*).	N/A
What worked well and opportunities for improvement		An open-ended survey question asking, “Please describe three likes and three dislikes from the program” will be used to assess general likes and dislikes towards the PA intervention.
Engagement motives and preferences for PA delivery	A closed-ended survey question asking, “Did you have a preference for intervention delivery?” will be used to assess preferences across response options of no preference, one-on-one delivery, and group-based delivery.	If students report a preference for intervention delivery, an open-ended survey question will ask students to describe reasons for their preference. An open-ended survey question asking, “Please briefly describe your reasons for wanting to participate in the intervention” will be used to assess engagement motives.
**Reach**		
	Percentage (%) of students eligible, % excluded, exclusion reasons, and student referral pathways including response options of on-campus health and well-being services, student accessibility services, social media advertisement, and other.	N/A
**Adherence**		
	Number of students who initiated the program, sessions completed, and completion rates.	Reasons for not initiating or completing the program will be recorded.

*Note*. PA = physical activity.

### Sample size

A simulation-based power analysis using the *simr* package in R was conducted [75]. This analysis estimated the likelihood of detecting a group-by-time interaction in a linear mixed model using restricted maximum likelihood. The simulated design included three groups (1:1 PA intervention, group PA intervention, and waitlist control), with primary and secondary outcomes assessed at three time points: pre-intervention (T1), post-intervention (T2), and follow-up (T3). The model included fixed effects for group, time, and their interaction, as well as a random intercept for students to account for within-subject correlations. A moderate treatment effect (*d* = .50) was applied at the post-intervention time point, and a smaller sustained effect (*d* = .20) was applied at follow-up. Simulated data included realistic between- and within-person variability, and the outcome variable was centered to aid model convergence. To reflect anticipated study conditions aligned with typical attrition in PA and mental health intervention research [76,77], the analysis included missing data at random in approximately 20% of observations, with a slightly higher dropout rate (30%) at the follow-up time point. All available data from each student will be used, aligning with an intention-to-treat analysis, and no ad hoc imputation will be performed, as researchers have suggested that linear mixed models without such imputation are more powerful than ad hoc strategies [78].

Based on 1,000 simulations, a total sample size of 114 students (~38 per group) yielded an estimated power of 98.10% (95% CI: 97.05, 98.85) to detect the group-by-time interactions using a Type II F-test with Satterthwaite degrees of freedom at α = .05. This high level of power reflects the modeled effect structure and supports the adequacy of the proposed sample size.

### Recruitment

Student recruitment and data collection began November 24, 2023, and is estimated to be completed by October 24, 2025, with results expected by January 24, 2026. Purposive and snowball sampling procedures will be used to recruit post-secondary students who are physically inactive and experiencing poor mental health. Post-secondary students will be recruited and referred to the intervention through the team’s research and professional networks (e.g., health and wellness and student support services; student life listservs; campus mental health listservs). Digital recruitment materials, including email templates and poster advertisements, will provide details about the purpose of the intervention, the procedures involved, eligibility criteria, and a link to the screening questionnaire. The purpose of this broad recruitment strategy is to enhance outreach and more effectively engage the diverse student population. Program reach will be examined to assess who is engaging with the intervention and how. These findings will offer valuable insights into potential disparities in uptake and help inform future strategies to improve accessibility, equity, and representation in program delivery. The screening questionnaire will be administered through REDCap [37] and will allow students to “sign up” up for the intervention by providing their email address and answering several questions to confirm eligibility. The program coordinator will contact eligible students to confirm their involvement in the study and to arrange an intake meeting.

### Statistical analyses

Prior to conducting the main analyses, descriptive statistics will be calculated for baseline demographic characteristics (e.g., gender, age, sexual orientation, ethnicity/race) and main study variables at each assessment point (T1, T2, and T3) across the trial arms. To assess baseline equivalence between trial arms, differences in demographic characteristics and main study variables at baseline will be examined using chi-square tests and one-way analysis of variance. Differences in dropout will also be examined based on demographic characteristics and baseline study variables using chi-square tests and independent samples t-tests. The absence of systematic differences in dropout would support the plausibility of the Missing At Random (MAR) assumption, which underlies the use of linear mixed effects modeling in handling missing data [36,78,79].

For the main analyses, linear mixed effects modeling will be used to examine intervention effects on the primary and secondary outcomes over time and model assumptions will be tested [36,79]. Model specifications are provided above for a priori sample size calculation purposes. Models will be estimated using restricted maximum likelihood with the *lme4* and *lmerTest* packages in R Version 2025.05.0 + 496. The intra-class correlation coefficient (ICC) will be reported to quantify the proportion of total variance in the outcome attributable to between-person differences, relative to within-person changes across timepoints. Significant interactions will be probed using within-group and between-group pairwise comparisons conducted with the *emmeans* package. *P* value and *95% CI* adjustments using Tukey method for comparing a family of 3 estimates will be used to adjust for multiple comparisons.

For the mixed methods process evaluation, quantitative closed-ended questions will be analyzed using descriptive statistics (means and standard deviations or frequency counts). Independent-samples t-tests and chi-square tests will be used to examine group differences between 1:1 and group delivery formats on process-related outcomes. Open-ended qualitative responses will be analyzed using thematic analysis [80]. An iterative coding process will be used, combining both an inductive (data-driven) and deductive (theory-informed) approach [81]. Findings from both quantitative and qualitative analyses will be integrated during interpretation in the discussion to provide a comprehensive understanding of student experiences and implementation processes.

### Ethical considersations

Any important protocol modifications (e.g., eligibility criteria, outcomes, analyses) to the protocol will be submitted to the REB for approval, updated in trial registries, and communicated to students (if applicable). All changes will be clearly documented and reported in trial publications. Student recruitment and data collection is ongoing (see student recruitment for study timeline) with plans for future implementation research and toolkit development, following completion of the current protocol. Written informed consent (See S1 Appendix) will be obtained from all students, and they will be provided with a thorough explanation of the study objectives, the voluntary nature of their participation, their right to withdraw, and the risks and benefits of the study. While there are minimal risks associated with participating in this research, there are potential emotional risks related to group vulnerabilities, particularly due to self-reported poor mental health, as well as the inherent physical risks associated with engaging in PA. To mitigate emotional risks, students will receive a resource sheet outlining accessible and free mental health services available on-campus and in the community after the intake meeting (see S2 Appendix). To mitigate physical risks, the PA sessions will be delivered by certified Sport and Recreation coaches who have received standard training in behavior change coaching and PA program delivery, and students will receive clearance for PA engagement using the PAR-Q^+^ [32]. In addition, bi-weekly meetings will be held with the research team and PA coaches throughout the intervention to ensure safety, address concerns, and support student well-being. All data will be collected, managed, and stored through the secure data capture platform REDCap [37]. Any identifiable information (i.e., email addresses, names) will be password protected and will only be used for program coordination purposes. For further information on data storage and confidentiality see S1 Appendix.

Adverse events will be identified through both spontaneous reports from students and formal solicited check-ins conducted by PA coaches during weekly sessions. Coaches will routinely ask about students’ physical and mental well-being during weekly session, with prompts provided in the behavior change coaching workbook. Coaches will report any concerns to the research team during regular bi-weekly coach check-ins. All adverse events will be reviewed for severity and monitored by the principal investigator. No formal provisions for ancillary or post-trial care are planned, as the intervention involves supervised PA and poses no greater risk than typical supervised PA offered in the community. Any serious adverse events will be reported to the University of Toronto REB in accordance with institutional policy.

### Knowledge dissemination

A knowledge mobilization plan to optimize dissemination to our intended audiences (i.e., sport and recreation professionals, mental health professionals, students, academic audiences) has been developed. Practically, the findings from the research trial will serve to inform prevention and intervention efforts for mental health on post-secondary campuses. The research team will collaborate with our knowledge-users in on-campus sport and recreation and student mental health to disseminate the findings through dedicated platforms including *Best Practices in Canadian Higher Education* and *NIRSA Leaders in College Recreation*. Moreover, guided by the AGREE II instrument [82], the research findings will inform the development of a toolkit, which will include educational resources for coach training, as well as program delivery considerations to guide future implementation research and practice. A project summary infographic will be distributed to all study participants and shared through university media and mental health outreach outlets. Lastly, to target academic audiences, research findings and subsequent studies will be published in open-access, peer reviewed journals. The findings will also be presented at international and national conferences in the discipline of exercise psychology (e.g., *North American Society for the Psychology of Sport and Physical Activity*), youth mental health (e.g., *International Association for Youth Mental Health*) and public health (e.g., *International Society for Physical Activity and Health)*. Due to ethical considerations for human research participant data, data and statistical code will only be available upon project completion (October 24, 2025) by request from the corresponding author, Dr. Catherine M. Sabiston (email: catherine.sabiston@utoronto.ca). The behaviour change coaching workbook used to guide the PA sessions will also be available by request from the corresponding author.

## Supporting information

S1 Checklist SPIRIT checklist. (PDF)

S1 Appendix Consent form. (PDF)

S2 Appendix Mental health resource sheet. (PDF)

## References

[1] Ontario University and College Health Association. Supporting the mental health of emerging adults in Ontario’s post-secondary system. Ontario University and College Health Association. 2017. https://bp-net.ca/wp-content/uploads/2023/06/Supporting-the-Mental-Health-of-Emerging-Adults-in-Ontario-Post-Secondary-System.pdf

[2] Tanner JL. Recentering during emerging adulthood: A critical turning point in life span human development. In: Arnett JJ, Tanner JL, editors. Emerging adults in America: Coming of age in the 21st century. Washington: American Psychological Association. 2006. p. 21–55.

[3] Jaworska N, De Somma E, Fonseka B, Heck E, MacQueen GM. Mental Health Services for Students at Postsecondary Institutions: A National Survey. Can J Psychiatry. 2016;61(12):766–75. doi: 10.1177/0706743716640752 27310230 PMC5564891

[4] Association for University and College Counseling Center Directors. Annual survey for reporting period July 1, 2022 through June 30, 2023. Association for University and College Counseling Center Directors. 2024. https://www.aucccd.org/assets/documents/Survey/2022-2023%20Annual%20Survey%20Report%20Public.pdf

[5] Dunley P, Papadopoulos A. Why is it so hard to get help? barriers to help-seeking in postsecondary students struggling with mental health issues: a scoping review. Int J Ment Health Addiction. 2019;17(3):699–715. doi: 10.1007/s11469-018-0029-z

[6] MacDonald H, Lisnyj K, Papadopoulos A. Facilitators and barriers highlighted by on-campus service providers for students seeking mental health services. CJHE. 2022;52(2):81–95. doi: 10.47678/cjhe.v52i2.189145

[7] Moghimi E, Stephenson C, Gutierrez G, Jagayat J, Layzell G, Patel C, et al. Mental health challenges, treatment experiences, and care needs of post-secondary students: a cross-sectional mixed-methods study. BMC Public Health. 2023;23(1):655. doi: 10.1186/s12889-023-15452-x 37020282 PMC10076091

[8] Cunningham CE, Zipursky RB, Christensen BK, Bieling PJ, Madsen V, Rimas H, et al. Modeling the mental health service utilization decisions of university undergraduates: A discrete choice conjoint experiment. J Am Coll Health. 2017;65(6):389–99. doi: 10.1080/07448481.2017.1322090 28511031

[9] Firth J, Siddiqi N, Koyanagi A, Siskind D, Rosenbaum S, Galletly C, et al. The Lancet Psychiatry Commission: a blueprint for protecting physical health in people with mental illness. Lancet Psychiatry. 2019;6(8):675–712. doi: 10.1016/S2215-0366(19)30132-4 31324560

[10] Firth J, Solmi M, Wootton RE, Vancampfort D, Schuch FB, Hoare E, et al. A meta-review of “lifestyle psychiatry”: the role of exercise, smoking, diet and sleep in the prevention and treatment of mental disorders. World Psychiatry. 2020;19(3):360–80. doi: 10.1002/wps.20773 32931092 PMC7491615

[11] Stubbs B, Vancampfort D, Hallgren M, Firth J, Veronese N, Solmi M, et al. EPA guidance on physical activity as a treatment for severe mental illness: a meta-review of the evidence and Position Statement from the European Psychiatric Association (EPA), supported by the International Organization of Physical Therapists in Mental Health (IOPTMH). Eur Psychiatry. 2018;54:124–44. doi: 10.1016/j.eurpsy.2018.07.004 30257806

[12] Stubbs B, Ma R, Schuch F, Mugisha J, Rosenbaum S, Firth J, et al. Physical activity and mental health: a little less conversation, a lot more action. J Phys Act Health. 2024;21(10):963–4. doi: 10.1123/jpah.2024-0404 39025470

[13] Jeftic I, Furzer BJ, Dimmock JA, Wright K, Boyd C, Budden T, et al. Structured exercise programs for higher education students experiencing mental health challenges: background, significance, and implementation. Front Public Health. 2023;11:1104918. doi: 10.3389/fpubh.2023.1104918 37181716 PMC10167056

[14] deJonge ML, Jain S, Faulkner GE, Sabiston CM. On campus physical activity programming for post-secondary student mental health: Examining effectiveness and acceptability. Mental Health and Physical Activity. 2021;20:100391. doi: 10.1016/j.mhpa.2021.100391

[15] Nesbitt AE, Collins KJ, Nalder E, Sabiston CM. Occupational outcomes of a physical activity intervention for post-secondary student mental Health. Can J Occup Ther. 2021;88(3):254–65. doi: 10.1177/00084174211021708 34132119

[16] Huang K, Beckman EM, Ng N, Dingle GA, Han R, James K, et al. Effectiveness of physical activity interventions on undergraduate students’ mental health: systematic review and meta-analysis. Health Promot Int. 2024;39(3):daae054. doi: 10.1093/heapro/daae054 38916148 PMC11196957

[17] Jetten J, Haslam C, von Hippel C, Bentley SV, Cruwys T, Steffens NK, et al. “Let’s get physical” - or social: The role of physical activity versus social group memberships in predicting depression and anxiety over time. J Affect Disord. 2022;306:55–61. doi: 10.1016/j.jad.2022.03.027 35301039

[18] Teychenne M, White RL, Richards J, Schuch FB, Rosenbaum S, Bennie JA. Do we need physical activity guidelines for mental health: What does the evidence tell us?. Mental Health and Physical Activity. 2020;18:100315. doi: 10.1016/j.mhpa.2019.100315

[19] Ryan RM, Deci EL. Self-determination theory: Basic psychological needs in motivation, development, and wellness. New York: The Guilford Press. 2017.

[20] Stevens M, Rees T, Coffee P, Steffens NK, Haslam SA, Polman R. A Social Identity Approach to Understanding and Promoting Physical Activity. Sports Med. 2017;47(10):1911–8. doi: 10.1007/s40279-017-0720-4 28349449 PMC5603625

[21] Donnelly S, Penny K, Kynn M. The effectiveness of physical activity interventions in improving higher education students’ mental health: A systematic review. Health Promot Int. 2024;39(2):daae027. doi: 10.1093/heapro/daae027 38563387 PMC10985680

[22] Curran GM, Bauer M, Mittman B, Pyne JM, Stetler C. Effectiveness-implementation hybrid designs: combining elements of clinical effectiveness and implementation research to enhance public health impact. Med Care. 2012;50(3):217–26. doi: 10.1097/MLR.0b013e3182408812 22310560 PMC3731143

[23] Glasgow RE, Lichtenstein E, Marcus AC. Why don’t we see more translation of health promotion research to practice? Rethinking the efficacy-to-effectiveness transition. Am J Public Health. 2003;93(8):1261–7. doi: 10.2105/ajph.93.8.1261 12893608 PMC1447950

[24] Moore GF, Audrey S, Barker M, Bond L, Bonell C, Hardeman W, et al. Process evaluation of complex interventions: Medical Research Council guidance. BMJ. 2015;350:h1258. doi: 10.1136/bmj.h1258 25791983 PMC4366184

[25] Vella SA, Aidman E, Teychenne M, Smith JJ, Swann C, Rosenbaum S, et al. Optimising the effects of physical activity on mental health and wellbeing: A joint consensus statement from Sports Medicine Australia and the Australian Psychological Society. J Sci Med Sport. 2023;26(2):132–9. doi: 10.1016/j.jsams.2023.01.001 36737260

[26] Ashdown-Franks G, DeJonge M, Arbour-Nicitopoulos KP, Sabiston CM. Exploring the feasibility and acceptability of a physical activity programme for individuals with serious mental illness: A case study. Qualitative Research in Sport, Exercise and Health. 2022;14(6):933–55. doi: 10.1080/2159676x.2021.2019098

[27] Schulz KF, Altman DG, Moher D. CONSORT 2010 Statement: Updated Guidelines for Reporting Parallel Group Randomized Trials. Ann Intern Med. 2011;154(4):291–2. doi: 10.7326/0003-4819-154-4-201102150-00017 21320945

[28] Chan A-W, Tetzlaff JM, Altman DG, Laupacis A, Gøtzsche PC, Krleža-Jerić K, et al. SPIRIT 2013 statement: defining standard protocol items for clinical trials. Ann Intern Med. 2013;158(3):200–7. doi: 10.7326/0003-4819-158-3-201302050-00583 23295957 PMC5114123

[29] de Jonge ML, Omran J, Faulkner GE, Sabiston CM. University students’ and clinicians’ beliefs and attitudes towards physical activity for mental health. Ment Health Phys Act. 2020;18:100316. doi: 10.1016/j.mhpa.2019.100316

[30] Orfaly RA, Frances JC, Campbell P, Whittemore B, Joly B, Koh H. Train-the-trainer as an educational model in public health preparedness. J Public Health Manag Pract. 2005;Suppl:S123-7. doi: 10.1097/00124784-200511001-00021 16205531

[31] Godin G. The Godin-Shephard leisure-time physical activity questionnaire. Health and Fitness J Canada. 2011;4(1). doi: 10.14288/hfjc.v4i1.82

[32] Warburton DER, Jamnik VK, Bredin SSD, Gledhill N. The physical activity readiness questionnaire for everyone (PAR-Q) and electronic physical activity readiness medical examination (ePARmed-X). Health and Fitness Journal of Canada. 2011;4(2):3–17. doi: 10.14288/hfjc.v4i2.103

[33] McFadden T, Fortier MS, Guérin E. Investigating the effects of Physical Activity Counselling on depressive symptoms and physical activity in female undergraduate students with depression: A multiple baseline single-subject design. Mental Health and Physical Activity. 2017;12:25–36. doi: 10.1016/j.mhpa.2017.01.002

[34] Kang M, Ragan BG, Park J-H. Issues in outcomes research: an overview of randomization techniques for clinical trials. J Athl Train. 2008;43(2):215–21. doi: 10.4085/1062-6050-43.2.215 18345348 PMC2267325

[35] Sil A, Kumar P, Kumar R, Das NK. Selection of Control, Randomization, Blinding, and Allocation Concealment. Indian Dermatol Online J. 2019;10(5):601–5. doi: 10.4103/idoj.IDOJ_149_19 31544090 PMC6743387

[36] Brown VA. An introduction to linear mixed-effects modeling in R. Adv Methods Pract Psychol Sci. 2021;4(1). doi: 10.1177/2515245920960

[37] Harris PA, Taylor R, Minor BL, Elliott V, Fernandez M, O’Neal L, et al. The REDCap consortium: Building an international community of software platform partners. J Biomed Inform. 2019;95:103208. doi: 10.1016/j.jbi.2019.103208 31078660 PMC7254481

[38] Michie S, Richardson M, Johnston M, Abraham C, Francis J, Hardeman W, et al. The behavior change technique taxonomy (v1) of 93 hierarchically clustered techniques: building an international consensus for the reporting of behavior change interventions. Ann Behav Med. 2013;46(1):81–95. doi: 10.1007/s12160-013-9486-6 23512568

[39] Lederman O, Suetani S, Stanton R, Chapman J, Korman N, Rosenbaum S, et al. Embedding exercise interventions as routine mental health care: implementation strategies in residential, inpatient and community settings. Australas Psychiatry. 2017;25(5):451–5. doi: 10.1177/1039856217711054 28585448

[40] Gaglio B, Phillips SM, Heurtin-Roberts S, Sanchez MA, Glasgow RE. How pragmatic is it? Lessons learned using PRECIS and RE-AIM for determining pragmatic characteristics of research. Implement Sci. 2014;9:96. doi: 10.1186/s13012-014-0096-x 25163664 PMC4243945

[41] Carney R, Bradshaw T, Yung AR. Physical health promotion for young people at ultra-high risk for psychosis: An application of the COM-B model and behaviour-change wheel. Int J Ment Health Nurs. 2016;25(6):536–45. doi: 10.1111/inm.12243 27432534 PMC6853191

[42] Cane J, Richardson M, Johnston M, Ladha R, Michie S. From lists of behaviour change techniques (BCTs) to structured hierarchies: comparison of two methods of developing a hierarchy of BCTs. Br J Health Psychol. 2015;20(1):130–50. doi: 10.1111/bjhp.12102 24815766

[43] Michie S, van Stralen MM, West R. The behaviour change wheel: a new method for characterising and designing behaviour change interventions. Implement Sci. 2011;6:42. doi: 10.1186/1748-5908-6-42 21513547 PMC3096582

[44] Hardy CJ, Rejeski WJ. Not What, but How One Feels: The Measurement of Affect during Exercise. Journal of Sport and Exercise Psychology. 1989;11(3):304–17. doi: 10.1123/jsep.11.3.304

[45] Svebak S, Murgatroyd S. Metamotivational dominance: A multimethod validation of reversal theory constructs. Journal of Personality and Social Psychology. 1985;48(1):107–16. doi: 10.1037/0022-3514.48.1.107

[46] Thornton JS, Frémont P, Khan K, Poirier P, Fowles J, Wells GD, et al. Physical activity prescription: a critical opportunity to address a modifiable risk factor for the prevention and management of chronic disease: a position statement by the canadian academy of sport and exercise medicine. Clin J Sport Med. 2016;26(4):259–65. doi: 10.1097/JSM.0000000000000363 27359294

[47] Frémont P, Fortier M, Frankovich RJ. Exercise prescription and referral tool to facilitate brief advice to adults in primary care. Can Fam Physician. 2014;60(12):1120–2, e591-2. 25500602 PMC4264809

[48] Day T, Tosey P. Beyond SMART? A new framework for goal setting. The Curriculum Journal. 2011;22(4):515–34. doi: 10.1080/09585176.2011.627213

[49] Borg G. Perceived exertion as an indicator of somatic stress. Scand J Rehabil Med. 1970;2(2):92–8. 5523831

[50] NEFF K. Self-Compassion: An Alternative Conceptualization of a Healthy Attitude Toward Oneself. Self and Identity. 2003;2(2):85–101. doi: 10.1080/15298860309032

[51] Bieleke M, Keller L, Gollwitzer PM. If-then planning. European Rev Social Psychol. 2020;32(1):88–122. doi: 10.1080/10463283.2020.1808936

[52] Forsyth DR. Recent advances in the study of group cohesion. Group Dynamics: Theory, Research, and Practice. 2021;25(3):213–28. doi: 10.1037/gdn0000163

[53] Regier DA, Kuhl EA, Kupfer DJ. The DSM-5: Classification and criteria changes. World Psychiatry. 2013;12(2):92–8. doi: 10.1002/wps.20050 23737408 PMC3683251

[54] Kroenke K, Spitzer RL, Williams JB. The PHQ-9: validity of a brief depression severity measure. J Gen Intern Med. 2001;16(9):606–13. doi: 10.1046/j.1525-1497.2001.016009606.x 11556941 PMC1495268

[55] Lorenzo A, Kthupi A, Liu W, Hamza C, Todorova AA, Kuburi S, et al. The mental health impact of the COVID-19 pandemic on post-secondary students: A longitudinal study. Psychiatry Res. 2023;327:115401. doi: 10.1016/j.psychres.2023.115401 37567112

[56] Sun Y, Fu Z, Bo Q, Mao Z, Ma X, Wang C. The reliability and validity of PHQ-9 in patients with major depressive disorder in psychiatric hospital. BMC Psychiatry. 2020;20(1):474. doi: 10.1186/s12888-020-02885-6 32993604 PMC7525967

[57] Spitzer RL, Kroenke K, Williams JBW, Löwe B. A brief measure for assessing generalized anxiety disorder: the GAD-7. Arch Intern Med. 2006;166(10):1092–7. doi: 10.1001/archinte.166.10.1092 16717171

[58] Bentley KH, Sakurai H, Lowman KL, Rines-Toth L, McKowen J, Pedrelli P, et al. Validation of brief screening measures for depression and anxiety in young people with substance use disorders. J Affect Disord. 2021;282:1021–9. doi: 10.1016/j.jad.2021.01.005 33601674 PMC7896042

[59] Byrd-Bredbenner C, Eck K, Quick V. GAD-7, GAD-2, and GAD-mini: Psychometric properties and norms of university students in the United States. Gen Hosp Psychiatry. 2021;69:61–6. doi: 10.1016/j.genhosppsych.2021.01.002 33571925

[60] Kessler RC, Andrews G, Colpe LJ, Hiripi E, Mroczek DK, Normand SLT, et al. Short screening scales to monitor population prevalences and trends in non-specific psychological distress. Psychol Med. 2002;32(6):959–76. doi: 10.1017/s0033291702006074 12214795

[61] Andrews G, Slade T. Interpreting scores on the kessler psychological distress scale (K10). Aust N Z J Public Health. 2001;25(6):494–7. doi: 10.1111/j.1467-842x.2001.tb00310.x 11824981

[62] Furukawa TA, Kessler RC, Slade T, Andrews G. The performance of the K6 and K10 screening scales for psychological distress in the australian national survey of mental health and well-being. Psychol Med. 2003;33(2):357–62. doi: 10.1017/s0033291702006700 12622315

[63] Chen S-P, Chang W-P, Stuart H. Self-reflection and screening mental health on Canadian campuses: validation of the mental health continuum model. BMC Psychol. 2020;8(1):76. doi: 10.1186/s40359-020-00446-w 32727614 PMC7391623

[64] Weatherson KA, Joopally H, Wunderlich K, Kwan MY, Tomasone JR, Faulkner G. Post-secondary students’ adherence to the Canadian 24-hour movement guidelines for adults: results from the first deployment of the canadian campus wellbeing survey (CCWS). Health Promot Chronic Dis Prev Can. 2021;41(6):173–81. doi: 10.24095/hpcdp.41.6.01 34164969 PMC8269782

[65] Veit CT, Ware JE Jr. The structure of psychological distress and well-being in general populations. J Consult Clin Psychol. 1983;51(5):730–42. doi: 10.1037//0022-006x.51.5.730 6630688

[66] Al Mutair A, Al Mohaini M, Fernandez R, Moxham L, Lapkin S, Ham-Baloyi WT. Psychometric testing of the mental health inventory in an Arabian context: cross-cultural validation study. Nurs Open. 2018;5(3):376–83. doi: 10.1002/nop2.149 30062032 PMC6056453

[67] Sutton A. Living the good life: A meta-analysis of authenticity, well-being and engagement. Personality and Individual Differences. 2020;153:109645. doi: 10.1016/j.paid.2019.109645

[68] Orpana HM, Lang JJ, Yurkowski K. Validation of a brief version of the social provisions scale using Canadian national survey data. Health Promot Chronic Dis Prev Can. 2019;39(12):323–32. doi: 10.24095/hpcdp.39.12.02 31825785 PMC6938275

[69] Lee RM, Robbins SB. Measuring belongingness: The Social Connectedness and the Social Assurance scales. Journal of Counseling Psychology. 1995;42(2):232–41. doi: 10.1037/0022-0167.42.2.232

[70] Godin G, Shephard RJ. A simple method to assess exercise behavior in the community. Can J Appl Sport Sci. 1985;10(3):141–6. 4053261

[71] Vani MF, Sabiston CM, Trinh L, Santa Mina D. Testing the associations between body image, social support, and physical activity among adolescents and young adults diagnosed with cancer. Front Psychol. 2022;12:800314. doi: 10.3389/fpsyg.2021.800314 35046877 PMC8761661

[72] Smith-Turchyn J, Sinclair S, O’Loughlin EK, Innes A, Vani MF, Beauchamp M, et al. A pilot randomized controlled trial of a virtual peer-support exercise intervention for female older adults with cancer. BMC Geriatr. 2024;24(1):887. doi: 10.1186/s12877-024-05495-z 39462335 PMC11515269

[73] Wierts CM, Rhodes RE, Faulkner G, Zumbo BD, Beauchamp MR. An online delivered running and walking group program to support low-active post-secondary students’ well-being and exercise behavior during the COVID-19 pandemic: a pilot randomized controlled trial. J Behav Med. 2024;47(6):935–50. doi: 10.1007/s10865-024-00516-z 39225842

[74] Tracey TJ, Kokotovic AM. Factor structure of the Working Alliance Inventory. Psychological Assessment: A Journal of Consulting and Clinical Psychology. 1989;1(3):207–10. doi: 10.1037/1040-3590.1.3.207

[75] Green P, MacLeod CJ. SIMR: an R package for power analysis of generalized linear mixed models by simulation. Methods Ecol Evol. 2016;7(4):493–8. doi: 10.1111/2041-210x.12504

[76] Vancampfort D, Sánchez CPR, Hallgren M, Schuch F, Firth J, Rosenbaum S, et al. Dropout from exercise randomized controlled trials among people with anxiety and stress-related disorders: A meta-analysis and meta-regression. J Affect Disord. 2021;282:996–1004. doi: 10.1016/j.jad.2021.01.003 33601745

[77] Stubbs B, Vancampfort D, Rosenbaum S, Ward PB, Richards J, Soundy A, et al. Dropout from exercise randomized controlled trials among people with depression: A meta-analysis and meta regression. J Affect Disord. 2016;190:457–66. doi: 10.1016/j.jad.2015.10.019 26551405

[78] Chakraborty H, Gu H. A mixed model approach for intent-to-treat analysis in longitudinal clinical trials with missing values. Research Triangle Park, NC. 2009. 30896910

[79] Schielzeth H, Dingemanse NJ, Nakagawa S, Westneat DF, Allegue H, Teplitsky C, et al. Robustness of linear mixed‐effects models to violations of distributional assumptions. Methods Ecol Evol. 2020;11(9):1141–52. doi: 10.1111/2041-210x.13434

[80] Braun V, Clarke V, Weate P. Using thematic analysis in sport and exercise research. Routledge Handbook of Qualitative Research in Sport and Exercise. 2021.

[81] Braun V, Clarke V. Reflecting on reflexive thematic analysis. Qualitative Research in Sport, Exercise and Health. 2019;11(4):589–97. doi: 10.1080/2159676x.2019.1628806

[82] Brouwers MC, Kho ME, Browman GP, Burgers JS, Cluzeau F, Feder G, et al. AGREE II: advancing guideline development, reporting and evaluation in health care. CMAJ. 2010;182(18):E839-42. doi: 10.1503/cmaj.090449 20603348 PMC3001530

