# Supplementary material for: One-on-one and group-based physical activity intervention compared to a waitlist control for post-secondary student mental health and social well-being: A 3-arm parallel randomized controlled trial protocol
Source: PLoS One. 2025 Aug 29;20(8):e0330851. doi: 10.1371/journal.pone.0330851 (PMC12396654; doi:10.1371/journal.pone.0330851)
Supplement: S1 Appendix — (PDF) [file pone.0330851.s002.pdf]

Letter of Informed Consent

Understanding on-campus physical activity programs for student mental health

INTRODUCTION

You are being invited to participate in a study exploring on-campus physical activity programs for post-secondary student mental health. You are invited to participate in this study because you have self-identified as: (1) being enrolled (either full-time or part-time) as a student at a Canadian post-secondary institution; (2) physical inactive; (3) having experienced poor mental health in the past month that has significantly affected your everyday functioning; and (4) able to attend weekly in-person exercise sessions at the University of Toronto, on the St. George campus. Experiences of poor mental health include experiences associated with poor mental health (not limited to a clinical diagnosis) that significantly affects your mood, thinking, and behaviour including symptoms of anxiety, depression, psychological distress and experiences of social isolation/loneliness, and difficulty coping with stress in a healthy way.

BACKGROUND

One-on-one (1:1) supervised exercise programs are supported and deemed effective and valuable by on-campus mental health professionals and mental health help-seeking students. On-campus physical activity programs may also be a valuable alternative approach for supporting student mental health and expanding the range of mental health programs available to students. While 1:1 program delivery models are supported as effective, we do not know if less resource-intensive delivery models, such as supervised groups, provide similar or unique benefits (e.g., regarding compliance, maintenance, mental health benefits) to 1:1 programs. Indeed, group-based exercise, compared to 1:1 exercise, may have unique benefits for cost-effectiveness, as well as for facilitating peer-to-peer social support and a sense of belonging among students.

PURPOSE OF THE STUDY

The purpose of this study is to assess the immediate (post-intervention) and follow-up (1-month) maintenance effects of 1:1 supervised exercise and group-based exercise in supporting post-secondary student mental health, a sense of belonging, social support, and exercise behaviour. The purpose will be achieved through a 3-arm randomized controlled trial, where your participation will involve being randomly assigned to receive 1:1 exercise, group-based exercise (consisting of small 3-8 person groups), or a 10-week waitlist. If you are assigned to the waitlist condition you will receive exercise training following completion of the 10-week waitlist (either group or 1:1 delivery depending on your preference).

STUDY PROCEDURES

If you agree to participate in this research, you will be asked to virtually sign this consent form. You will then be directed automatically to a screening questionnaire to confirm study eligibility and to obtain your email address for scheduling purposes only. Once the screening questionnaire is complete, a program coordinator will contact you within 48 hours to confirm your participation in the study. If you are eligible to participate, the next step is to schedule an in-person intake meeting with the program coordinator. The intake meeting will be 1:1 and held in

the Mental Health and Physical Activity Research Centre in the Athletic Centre at the University of Toronto, St. George Campus (55 Harbord Street). Your involvement in the study will include:

- **Participation in a 6-week exercise program.** You will be randomly assigned to receive:
  (1) 1:1 and individualized exercise training
  (2) Group-based exercise (consisting of small 3-8 person groups); or
  (3) A 10-week waitlist. If you are assigned to the waitlist condition you will receive exercise training following completion of the 10-week waitlist.
- The 6-week exercise program will involve weekly 1-hour sessions that consist of: (1) 30-minutes of behaviour change coaching (e.g., goal setting, action-planning, brainstorming strategies to overcome barriers to engaging in exercise); and (2) 30-minutes of supervised exercise.
- **Completion of ~ 30-minute self-report questionnaires** at baseline, post-intervention (6-weeks), and 1-month follow-up, following completion of the post-intervention questionnaire. Questionnaires will assess demographic characteristics, mental health, social support, feelings of belonging, exercise behaviour, and program satisfaction.

All exercise sessions will be delivered by a certified Sport and Recreation coach at the University of Toronto. You will be matched with the same certified coach for the entire duration of the program. Throughout your 6-week involvement in the study, you will not receive financial compensation beyond the weekly 1-hour certified coach-led exercise sessions. Following completion of the 6-week program, you will be compensated $25 for completing the 1-month 30-minute follow-up survey.

POTENTIAL BENEFITS AND RISKS

The current study extends existing work on exercise programming for mental health within a post-secondary context and aims to establish replicable and feasible approaches for delivering university-based exercise programs for student mental health. You will benefit from a unique opportunity to receive behaviour change coaching and exercise training from a certified Sport and Recreation Coach at the University of Toronto. If successful, the combined behaviour change coaching and supervised exercise training will provide benefits for overcoming barriers to engaging in exercise, promoting exercise behaviour change, and for promoting social and emotional well-being. If successful, this research will also benefit the post-secondary community. Implementing a program like this could expand the range of mental health and well-being programs available to students, with a focus on exercise and behaviour change.

While there are minimal risks to participating in the study, some questions in the surveys may potentially provoke negative emotions or may elicit some uncomfortable thoughts and/or feelings. You are not required to answer any questions that you are uncomfortable answering, and you will be given contact information for mental health resources and support. There may also be the possibility of injury while exercising. Serious risks are rare but include cardiac events and musculoskeletal injuries. To reduce the risk of sustaining any injuries, the exercise sessions will be delivered by certified Sport and Recreation coaches. You are not required to perform any exercise that is deemed uncomfortable and will be instructed to stop engaging in exercise if you experience sharp pain, nausea, dizziness or light-headedness. The exercise sessions will also be tailored to your interests and needs in the 1:1 exercise sessions. In the group exercise sessions,

you will be provided with options to regress (make the exercise easier) or progress (make the exercise harder) the instructed exercise, depending on individual interests and exercise experience.

CONFIDENTIALITY

All information collected for this study will be kept strictly confidential. The information will be stored electronically in secure, password-protected folders on the drive within the Mental Health and Physical Activity Research Centre at the University of Toronto until it is permanently discarded upon completion of this project. The data will be stored securely for five years after the scientific reports on this research project are published. At this point, the electronic copies of data (including screening questionnaire data and pre-post-and-follow-up data) and electronic consent forms will be erased. All data will be deidentified and coded by participant ID to ensure confidentiality of the data. The results of this study may be published and presented at conferences and in academic journals, but all data will be anonymized and presented as aggregate-level data to maintain confidentiality.

VOLUNTARY PARTICIPATION AND/OR WILTHDRAWAL

Participation in this study is completely voluntary and has no ties to or impact on your current or future class activities, mental health services or athletic programming opportunities, future research involvement, or your standing at the University of Toronto in any way. You may refuse to participate or may choose to discontinue with the study at any time without consequence. If you choose to withdraw from the study, you may contact the student investigator (melissa.dejonge@utoronto.ca) or the program email (movehappyu@utoronto.ca) to inform the researchers of your decision. After you have informed the researchers of your decision, you will be contacted and notified that all data has been discarded. If analysis has taken place, withdrawal will not be possible. Data analysis is expected to begin by October 25, 2025.

QUESTIONS AND CONTACT INFORMATION

If you have any questions or concerns regarding this study or desire further information, please contact the student investigator, Melissa deJonge, at melissa.dejonge@mail.utoronto.ca. Dr. Catherine Sabiston, the principal investigator for this project, can be reached at catherine.sabiston@utoronto.ca. Participants can contact the Office of Research Ethics at the University of Toronto at ethics.review@utoronto.ca or 416-946-3273, if you have questions or concerns about your rights as participants in research. **Ethics approved protocol #:** 45228

DECLARATION OF CONSENT

I have read the content of this consent form, and I agree to participate in this study.
