# Supplementary material for: One-on-one and group-based physical activity intervention compared to a waitlist control for post-secondary student mental health and social well-being: A 3-arm parallel randomized controlled trial protocol
Source: PLoS One. 2025 Aug 29;20(8):e0330851. doi: 10.1371/journal.pone.0330851 (PMC12396654; doi:10.1371/journal.pone.0330851)
Supplement: S2 Appendix — (PDF) [file pone.0330851.s003.pdf]

Mental Health Resources on University of Toronto Campus

The Health & Wellness Centre

https://www.studentlife.utoronto.ca/hwc
416-978-8030
700 Bay Street

The Health & Wellness Centre provides the same services as your family physician. Students who have registered and paid fees at the University of Toronto are eligible for services.

Same-day counselling: Counsellors are available for same-day, single-session counselling for help with issues like roommate conflict, exam stress, relationship problems, sadness or nervousness and more. Appointments are limited and will be scheduled on a first-come, first-served basis. Counsellors can be found in Room 232 on Tuesdays and Thursdays, 1 – 4 p.m.

Graduate Counselling Services

https://www.sgs.utoronto.ca/resources-supports/graduate-wellness-services-at-sgs/
416-978-8030
63 St. George Street
School of Graduate Studies,

Two full-time Wellness Counsellors from the Health and Wellness Centre on St. George Campus offer brief counselling services tailored to the challenges presented by graduate-level university life. The focus of counselling is on identifying strengths, acquiring resiliency, and building coping skills.

SafeTALK

email: health.promotion@utoronto.ca

SafeTALK is a 4-hour training designed to ensure that people with thoughts of suicide are connected to helpers who are prepared to provide first-aid interventions. SafeTALK is free for all students currently studying at University of Toronto.

Physical Activity and Exercise on University of Toronto Campus

UofT Sport and Recreation

https://kpe.utoronto.ca/sport-recreation/sport-rec-university-toronto

Sport & Rec offers sport and physical activity programs to the University of Toronto community on the downtown campus, as well as the local community. There are many drop-in programs that students can register in throughout the year.

Hart House

https://harthouse.ca/

Open 365 days a year and located downtown on the University of Toronto campus, the Hart House Fitness Centre features a pool, squash courts, multi-level fitness facilities, suspended indoor track and a variety of spaces for everything from basketball to archery. Eligible U of T students are automatic members, but anyone can join. Learn more about membership options and special discounts.

Body Image and Nutrition Counseling

National Eating Disorder Information Centre

https://nedic.ca/health-promotion-prevention/
416-340-4156

NEDIC provides information, resources, referrals and support to Canadians affected by eating disorders through their toll-free helpline and instant chat. Outreach and education programming is available online and in the Greater Toronto Area, focusing on awareness and the prevention of eating disorders.

Sheena's Place

www.sheenasplace.org
416-927-8900

Support groups are for those affected by eating disorders, including groups for individuals who are affected themselves as well as family members, partners or friends of loved ones who have eating disorders. There are no fees, groups run for 8 – 10 weeks at a time 4 times a year. Participants must register during one of our registration periods. ** You must be 17 years of age or older to attend groups at Sheena's Place.

Bulimia Anorexia Nervosa Association (BANA)

https://bana.ca/
519-969-2112

BANA is a not-for-profit, registered charity, community-based providing specialized treatment, education and support services for individuals affected by eating disorders. Refer to the website for extensive information on resources, workshops and more information.

National Initiative for Eating Disorders (NIED)

https://www.nied.ca/
613-795-8414

NIED provides support and hope for Eating Disorder individuals and families. NIED is working with caregivers, healthcare and social services providers to empower and help parents, families, individuals (including in rural and remote locations) to recognize early signs and know how to seek help

Community Resources

My Student Support Program (My SSP)

1-844-451-9700. Outside of North America, call 001-416-380-6578.

My Student Support Program" or My SSP, provides University of Toronto students with immediate and/or ongoing confidential, 24-hour support for any school, health, or general life concern at no cost to students. You can call or chat with a counsellor directly from your phone whenever, wherever you are for a range of concerns. Students who use My SSP still have access to existing campus and community mental health services; My SSP is an additional support service. Download the app on Apple Store or Google Play.

Good2Talk Helpline: 1-866-925-5454
Assaulted Women's Helpline: 416-863-0511
Gerstein Crisis Centre: 416-929-5200
Toronto Distress Centre: 416-408-4357
Drug and Alcohol Helpline: 1-800-565-8603
Mental Health Helpline: 1-866-531-2600
Ontario Problem Gambling Helpline: 1-888-230-3505
Toronto Rape Crisis Centre: 416-597-8808http://www.awhl.org/

If you are looking for Information & Referral services in Ontario:

ConnexOntario offers province-wide information and referral services for those experiencing mental health issues, drug or alcohol addiction, or problem gambling. You can visit their website at: www.connexontario.ca or you can call the following number which operates 24 hours a day, 7 days a week: 1-800-531-2600.

- **Mental Health Helpline:** 1-866-531-2500
- **Drug and Alcohol Helpline:** 1-800-565-8603
- **Ontario Problem Gambling Helpline:** 1-888-230- 3505

How do you know if you need help?

- You find yourself feeling overwhelmed by feelings of anger or despair, and you cannot enjoy life anymore
- You used to be healthy, but now you are always feeling a bit sick and you are missing more and more time from work.
- Your finances are out of control, and you are worried about being able to pay the next month's rent or mortgage payment
- You cannot "get over" the death of someone you loved very much
- There is too much conflict at home. You are afraid your marriage may break up
- You are drinking too much or having some other kind of drug problem
- You are feeling suicidal
